# Prevalence of mental disorders among Norwegian college and university students: a population-based cross-sectional analysis

**DOI:** 10.1016/j.lanepe.2023.100732

**Published:** 2023-09-19

**Authors:** Børge Sivertsen, Ann Kristin Skrindo Knudsen, Benedicte Kirkøen, Jens C. Skogen, Bengt Oscar Lagerstrøm, Kari-Jussie Lønning, Ronald C. Kessler, Anne Reneflot

**Affiliations:** a Department of Health Promotion, Norwegian Institute of Public Health, Bergen, Norway; b Department of Research & Innovation, Helse-Fonna HF, Haugesund, Norway; c Department of Disease Burden, Norwegian Institute of Public Health, Bergen, Norway; d Department of Mental Health and Suicide, Norwegian Institute of Public Health, Oslo, Norway; e Centre for Alcohol and Drug Research (KORFOR), Stavanger University Hospital, Stavanger, Norway; f Centre for Evaluation of Public Health Measures, Norwegian Institute of Public Health, Oslo, Norway; g Division for Social Surveys, Department for Methodology and Data Collection, Statistics Norway, Oslo, Norway; h The Student Welfare Organization in Oslo and Akershus (SiO), Oslo, Norway; i Modum Bad Psychiatric Hospital, Vikersund, Norway; j Department of Health Care Policy, Harvard Medical School, Boston, MA, USA

**Keywords:** Diagnostic interview, Epidemiological survey, College and university students, Mental disorders, Prevalence

## Abstract

**Background:**

Self-report data indicate a sharp increase in mental health problems among college and university students in recent years, but accurate prevalence estimates of mental disorders are lacking. The current study used a validated psychiatric diagnostic survey, developed into a self-administered electronic version, to examine the prevalence of common mental disorders in a large national sample of college and university students in Norway.

**Methods:**

Participants (aged 18–35 years) from the national Students’ Health and Wellbeing (SHOT) Study in 2022 were recruited to a follow-up online survey of mental disorders from January to February 2023 (n = 10,460). Current (30-days), 12-months and lifetime prevalence of common mental disorders were examined using a newly developed self-administered electronic version of the Composite International Diagnostic Interview (CIDI 5.0).

**Findings:**

The prevalence of a current mental disorder was high for both women (39.7% [2737/6886], 95% CI 38.6–40.9) and men (25.7% [751/2918], 95% CI 24.2–27.4). The most common disorders were major depressive episode (females 17.1% [1250/7329] and males 10.8% [331/3059]) and generalized anxiety disorder (females 16.0% [1157/7221] and males 8.2% [250/3032]), while 5.6% [387/6948] and 7.7% [228/2963] of the females and male students, respectively, fulfilled the criteria for an alcohol use disorder. The prevalence estimates for 12-month and lifetime were, as expected, even higher.

**Interpretation:**

The findings suggest an alarmingly high prevalence of several mental disorders among Norwegian college and university students. Implications and potential methodological and contextual explanations of these findings are discussed.

**Funding:**

10.13039/501100017488Norwegian Ministry of Education and Research.

**Research in context**

**Evidence before this study**

We searched APA PsycInfo and OVID Medline with the search terms mental disorders, anxiety disorders, mood disorders, depressive disorder, panic disorder, phobic disorder, alcohol disorder, substance disorder or variations of these search terms for systematic reviews in English published from January 1st 2013 until April 1st 2023. The search returned 6 systematic reviews describing studies in which the great majority relied on brief survey questionnaires or screening questions assessing symptoms of anxiety and/or depression. Only one systematic review included two studies with diagnostic interviews.^1^ A French study based on data from the WHO Composite International Diagnostic Interview -Short Form (CIDI) collected in 2005––2006 found 12 months prevalence of major depressive disorder, anxiety disorder and substance use disorder to be 8.9%, 15.7% and 8.1% respectively.^2^ A Chinese study based on data from the WHO CIDI 3.0 collected in 2007 found lifetime, 12-month and 30-day prevalence of neurotic disorders to be 25.6%, 15.7% and 6.8% respectively.^3^

**Added value of this study**

The present study is based on data from a newly developed electronic self-administered version of a standardized and validated psychiatric interview (CIDI 5.0). This approach enabled the recruitment of a large national sample of college and university students. Based on the 10,460 students (conditional response rate 63.7%) who completed the survey, the current study found an alarmingly high prevalence of several mental disorders. Overall, we found that 40% of the female students and 26% of the male students had a current mental disorder, and the 12-month prevalence was even higher (57% and 43%, respectively). The lifetime prevalence rates among female and male students were 67% and 54%, respectively. The timing of the study is important, as it was conducted more than a year after the removal of the many restrictions caused by the COVID-19 pandemic.

**Implications of all the available evidence**

The majority of reviewed studies suggest that colleges and universities worldwide are facing a very high prevalence of mental health problems among their student populations, with estimates substantially surpassing those observed in the general population. The present study provides further evidence of a disturbing trend; that a considerable part of college and university students are suffering from a mental disorder severe enough to require intervention. While this self-administered questionnaire version of CIDI has yet to be validated and compared against prevalence estimates obtained from face-to-face interviews, it is unlikely that administration mode alone can explain the findings. Given the negative consequences of untreated mental disorders, these findings call for both policy makers and educational institutions to sufficiently scale up their support mechanisms.

## Introduction

Colleges and universities across the world are facing a worrying increase in the number of students who suffer from mental health problems, and estimates are substantially higher than those found in the general population.^4^ Recent systematic reviews of anxiety and depression have estimated a pooled 12-month prevalence of 25–30% among college and university students.^3^, ^4^, ^5^, ^6^ However, most studies in this field have either used brief survey questionnaires or only the screening questions of diagnostic interviews when assessing mental health problems.^7^^,^^8^ Very few studies have used structured diagnostic interviews, which is considered as the gold standard for diagnosing mental disorders,^9^^,^^10^ as they are both costly and time consuming to conduct. One notable exception is the Dutch Nemesis studies of mental disorders from 2019 to 2022, which showed that 35–40% of adults aged 18–34 fulfilled the criteria for a mental disorder in the past 12 months, of which 20% had an anxiety disorder and 13–14% had a mood disorder.^11^^,^^12^

Being a crucial transitional period, young adulthood is a particularly important age cohort to focus on for several reasons. First, most mental disorders have an onset in late adolescence and early adulthood, with 60–70% of all lifetime mental disorders displaying before the age of 25 years.^13^ Second, data from the OECD show that in the last two decades, the proportion of young adults with tertiary education has increased from 27% to 48%, and the number is still rapidly growing.^14^ The increase in tertiary education attainment indicates an important transformation in the educational landscape and socioeconomic segmentation of young adults. Third, the latest studies assessing mental disorders among students are now more than 15 years old,^2^^,^^3^ and accurate and up-to-date prevalence estimates for college and university students are essential to both educational institutions and student welfare organizations to optimize their health services planning, resource allocation, and research priorities.^15^ And finally, the impact of mental illness on subsequent educational, social, and economic outcomes is devastating, with mental illness being both persistent^16^ and a main cause of dropout from higher education^17^ and suicide in young adults.^18^ Therefore, detecting mental disorders among college and university students provides an excellent opportunity for addressing the substantial burden of early-onset mental disorders. Moreover, the initial phase of the COVID-19 pandemic showed a sharp increase in levels of mental health problems, especially in young adults.^19^, ^20^, ^21^ There is clearly a need for new estimates of mental disorders based on data collected after the lifting of the many imposed restrictions and lockdowns.

The current study employed a newly developed electronic self-administered version of the World Health Organization (WHO) Composite International Diagnostic Interview, fifth version (CIDI 5.0), developed for the WHO World Mental Health (WMH) Surveys.^22^ This is the first time a validated psychiatric interview has been used in a self-administered manner, circumventing the traditional barriers associated with structured diagnostic interviews, while at the same time enabling the inclusion of a large-scale sample. The main objective of this paper was to determine the prevalence of common mental disorders (depressive episode, anxiety disorders, substance use disorders) using a large national sample of college and university students in Norway.

## Methods

This present report complies with the STROBE statement. The survey is registered at ClinicalTrials.gov (identifier: NCT05731102) and was a collaboration between the Norwegian Institute of Public Health (NIPH), Statistics Norway and the three largest student welfare organizations in Norway. The study was approved by the Regional Committee for Medical and Health Research Ethics in Western Norway (no. 2022/326437).

### Setting and participants

The base study population of the current study stems from the SHOT study (*Students’ Health and Wellbeing Study*), a large national survey of students enrolled in higher education in Norway. Four main surveys have been completed since 2010. The current study is based on the most recent wave, conducted in 2022. Details of SHOT have been published elsewhere,^23^ but in brief SHOT2022 was a comprehensive survey of several domains of health and lifestyle factors, including psychological distress, suicidality, life satisfaction, loneliness, sleep problems, sexual harassment, pain, physical exercise, use and attitudes towards alcohol and drugs, as well as several demographic and educational parameters. SHOT2022 was distributed electronically through a web-based platform, and was conducted between February 8 and April 19, 2022, inviting all full-time Norwegian students pursuing higher education, both in Norway and abroad. Students were approached by both email and SMS, and all but a few welfare organizations and educational institutions had information campaigns to help make their students aware of the study. In all, 169,572 students fulfilled the inclusion criteria, of whom 59,544 students completed the online questionnaires (after being sent two reminders), yielding a response rate of 35.1%. Using aggregated data obtained from the Norwegian State Educational Loan Fund, we found that the response rates were relatively similar across the 4 health regions in Norway, ranging from 32.1% to 37.5%. Of these, 53,362 students were between 18 and 35 years, which was the inclusion criterium for the current study.

When consenting to participate in the SHOT2022, students were also asked to indicate if they wished to be invited to a follow-up study of mental disorders, of whom 26,311 consented. To approximate a similar sex distribution as in the base study population, comparatively more males than females were invited to take part in the CIDI study, yielding an invited sample of 16,418 students (who were still officially registered as students in January 2023). However, as relatively fewer males consented to being contacted for a follow-up study, a larger proportion of females (70.4%) than males received an invitation to the CIDI. The CIDI study was conducted between January 24 and February 6, 2023, approximately 12 months after the SHOT2022.

### Instruments

#### Sociodemographic information

Data regarding the participants’ age and sex were extracted from their 11-digit Norwegian national identity number. Age was used both as a continuous and categorical variable. Additional background information was obtained by linking the CIDI study with the SHOT2022 study. In the SHOT2022, participants were asked about their relationship status (with the following response options ‘single’, ‘boy-/girlfriend’, ‘cohabitant’, ‘married’/“registered partner”). Financial difficulties were assessed by asking if the student during the last 12 months experienced difficulties affording costs of living (such as for food, transportation and accommodation; ‘never’, ‘rarely’, ‘sometimes’, ‘often’). Participants were also asked if either the student or his/her parents were born outside Norway, and their accommodation status was assessed and coded as ‘living alone’, ‘living with partner’, ‘living with friends’, or ‘living with parents’). Finally, the participants indicated the educational level of their parents as either having completed primary education, secondary education, or college/university education.

#### Mental disorders: the CIDI

A newly developed self-administered electronic version of the World Health Organization (WHO) Composite International Diagnostic Interview, fifth version (CIDI 5.0)¸ developed for the WHO World Mental Health (WMH) Surveys, was used for the data-collection.^22^ This self-administered electronic version was written in Blaise 5.4, a software tool designed to collect survey data. Blaise is used by several national statistics agencies in Europe, and Statistics Norway administered the Norwegian translation of the CIDI used in the current study. Statistics Norway also conducted the data collection.

CIDI 5.0 is a standardized interview assessing 30-days, 12 months and lifetime prevalence for several mental and substance use disorders according to diagnostic criteria in the Diagnostic and Statistical Manual of Mental Disorders 5th edition (DSM-5).^24^ CIDI 5.0. has good concordance with diagnostic instruments such as the Structured Clinical Interview for DSM-IV (SCID)^25^ and Schedules for Clinical Assessment in Neuropsychiatry (SCAN).^26^ The Norwegian version of the CIDI is based on the official Norwegian translation of CIDI. 5.0, as described in a previous study protocol publication.^27^

*Current mental disorder* was defined as presence of a mental disorder during the 30 days before study. We also report 12-months and *lifetime* prevalence of mental disorders. The following mental disorders were included in this variable: major depressive episode, generalized anxiety disorder, panic disorder, specific phobia, agoraphobia, social anxiety disorder, alcohol use disorder and drug use disorder. Operationalization of diagnoses was based on algorithms developed for CIDI 5.0 in the WMH Surveys Initiative and can be obtained upon request to the WMH or the authors.

#### Mental health problems

Mental health problems in the 14 days before survey were assessed by the widely used *Hopkins Symptoms Checklist* (HSCL-25),^28^ derived from the 90-item Symptom Checklist (SCL-90), a screening tool designed to detect symptoms of anxiety and depression. An investigation of the factor structure based on the SHOT2014 dataset showed that a unidimensional model had the best psychometric properties in the student population and not the original subscales of anxiety and depression.^29^ Details on development of mental health problems in the SHOT waves were recently published by Knapstad and colleagues.^30^ Students completed the HSCL-25 both in the SHOT2022 study, as well as in the CIDI follow-up study. In this present report, the HSCL-25 was used to examine the representativeness of the CIDI sample.

### Statistical analyses

All analyses used unweighted data, as the estimates are presented separately for male and female students, and the age- and sex distribution did not, or only marginally, differ from those of the base population of students. Also, we had little accurate information on a population level, which could form the basis for potential weighting. First, we calculated descriptive and clinical characteristics (age, sex, marital status, financial difficulties, country of birth, accommodation status, parental education, and HSCL-25 score) of the responders and non-responders of the CIDI study, and the total SHOT2022 sample. Statistical comparisons were made between CIDI responders vs. CIDI non-responders and CIDI responders vs. SHOT2022, using Chi-squared test (for categorical variables) or independent samples t-tests (for continuous variables). Between-group effect sizes (pooled standard deviation SD) were calculated for HSCL-25 using Cohen's *d* formula. Unweighted prevalence estimates, including 95% confidence intervals, were then calculated for 30-days, 12-month, and lifetime disorders. The CIDI includes independent diagnostic sections, and the specific prevalence estimates were based on participants with valid responses on the corresponding sections. A total of 10,460 participants had valid responses on at least one diagnostic section, and hence, the number of responses differs between the sections. The overall prevalence estimates (“any mental disorders”) are based on all participants with either at least one positive diagnosis or who had completed the full CIDI survey. Stata 17 SE was used for all analyses, while the tables were produced using gtsummary 1.7.1^31^ and R version 4.2.2.

### Role of the funding source

The funders had no role in study design, data collection, data analysis, data interpretation, or writing of the report. All authors had full access to all data in the study. Submission was approved by all co-authors. For the purpose of open access, the author has applied a CC BY public copyright licence to any Author Accepted Manuscript (AAM) version arising from this submission.

## Results

### Sample characteristics and representativeness

**Fig. 1:**
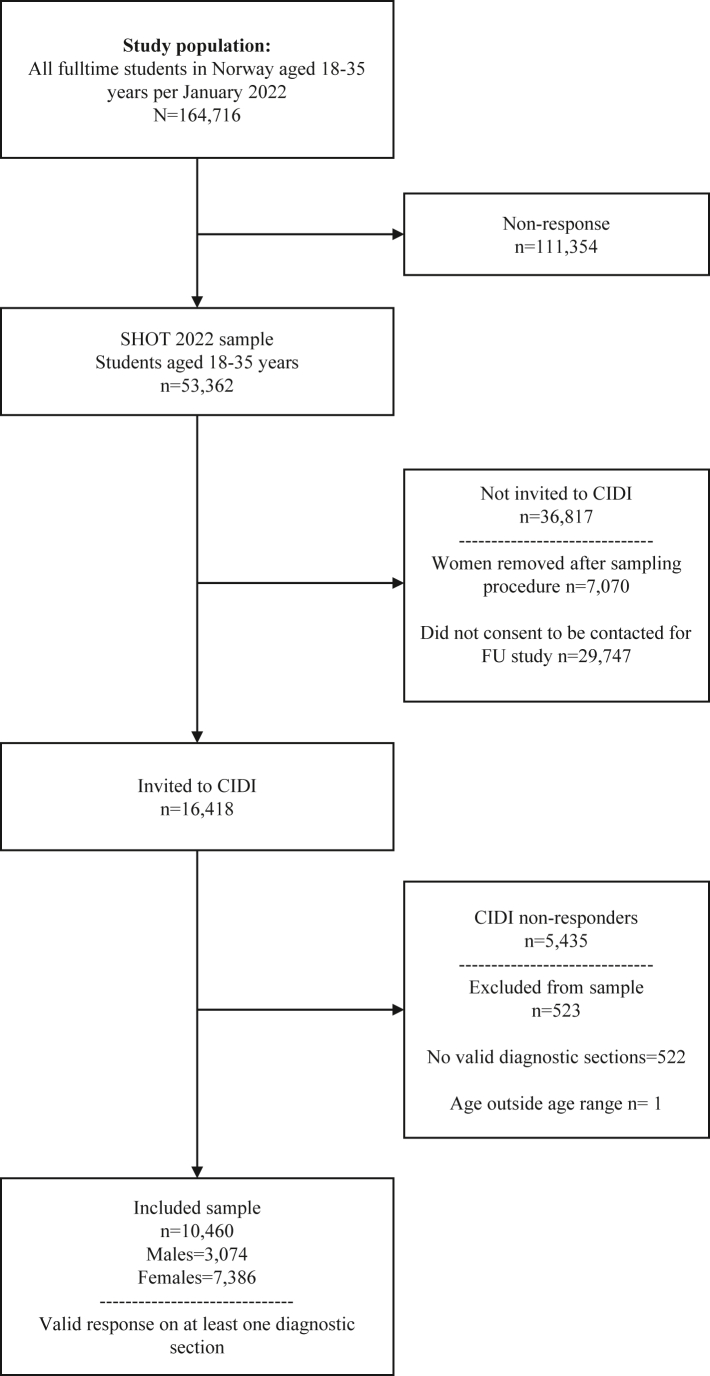
Flow-chart of survey participation process.

Fig. 1 details the participation process. A total of 10,460 students had a valid response on at least one of the CIDI diagnostic sections (conditional response rate 63.7%). The key sociodemographic and clinical characteristics of the students who completed the CIDI study, as well as the original SHOT2022 study are shown in Table. The sample of CIDI responders had a mean age of 24 years, were predominantly female and of Norwegian ethnicity, about half of the students reported being single. Approximately one in four students reported having financial difficulties “often” or “sometimes”. The parental education level of the participants was generally high. As also detailed in the Table 1, the sociodemographic characteristics among the participants who completed the CIDI study were largely consistent with the overall SHOT2022 study, with the exception of sex (females comprised 70.6% vs. 66.4% of the CIDI and SHOT2022 samples, respectively). As also detailed in Table 1, students who did *not* respond on the CIDI study, but were invited, did not differ significantly to CIDI responders across most sociodemographic factors, with the exception of having somewhat more financial difficulties, as well as having less educated parents.

**Table 1:** Sociodemographic and clinical characteristics in 2022 of the CIDI responders, CIDI non-responders and the overall SHOT2022 sample.

Characteristic	CIDI responders (n = 10,460)	CIDI non-responders (n = 5958)	p-value^b^	SHOT2022^a^ (n = 53,362)	p-value^b^
Age, mean (SD)	24.03 (3.28)	23.97 (3.24)	0.24	23.98 (1.85)	0.14
Age group, % (n)			0.74		0.76
18–22 years	45.8 (4789)	45.9% (2733)		46.3 (24,709)	
23–25 years	32.7 (3418)	33.4% (1990)		32.8 (17,504)	
26–35 years	21.5 (2253)	20.7% (1235)		20.9 (11,149)	
Sex, % (n)			0.97		<0.001
Women	70.6 (7386)	70.4 (4196)		66.4 (35,423)	
Men	29.4 (3074)	29.6 (1762)		33.6 (17,939)	
Marital status, % (n)			0.16		0.70
Single	51.2 (5359)	50.3 (2994)		51.0 (27,197)	
Boy-/girlfriend	22.4 (2343)	23.7 (1414)		22.8 (12,152)	
Cohabitant	22.7 (2375)	22.5 (1340)		22.6 (12,058)	
Married/registered partner	3.3 (345)	3.0 (178)		3.1 (1667)	
*Missing*	*0.4 (38)*	*0.5 (32)*		*0.5 (288)*	
Financial difficulties, % (n)			0.004		0.14
Never	55.1 (5766)	51.1 (3046)		54.0 (28,801)	
Rarely	20.1 (2106)	20.3 (1207)		20.8 (11,111)	
Sometimes	18.6 (1941)	20.3 (1206)		18.5 (9898)	
Often	5.8 (609)	8.0 (475)		6.1 (3245)	
*Missing*	*0.4 (38)*	*0.4 (24)*		*0.6 (307)*	
Self and/or parent(s) born abroad, % (n)			0.17		0.34
Born in Norway	81.2 (8491)	80.4 (4792)		80.1 (43,052)	
Born outside Norway	10.0 (1043)	10.9 (651)		10.4 (5541)	
*Missing*	*8.9 (926)*	*8.6 (515)*		*8.9 (4769)*	
Accommodation status, % (n)			0.66		0.37
Living alone	20.2 (2115)	20.3 (1212)		19.6 (10,442)	
Living with partner	27.0 (2824)	26.2 (1564)		26.7 (14,221)	
Living with friends	44.4 (4640)	45.1 (2684)		44.7 (23,838)	
Living with parents	8.1 (845)	8.1 (480)		8.6 (4591)	
*Missing*	*0.3 (36)*	*0.3 (18)*		*0.5 (270)*	
Maternal education, % (n)			0.04		0.50
Primary	4.4 (457)	5.3 (313)		4.5 (2407)	
Secondary	27.3 (2857)	27.6 (1646)		27.6 (14,707)	
College/university	65.6 (6858)	64.2 (3827)		64.3 (34,326)	
*Missing*	*2.8 (288)*	*2.9 (172)*		*3.6 (1992)*	
Paternal education, % (n)			0.02		0.52
Primary	5.7 (599)	6.8 (406)		6.0 (3182)	
Secondary	35.2 (3678)	24.9 (2078)		35.1 (18,735)	
College/university	54.2 (5674)	52.8 (3145)		53.3 (28,446)	
*Missing*	*4.9 (509)*	*5.5 (329)*		*5.6 (2999)*	
HSCL-25, Mean (SD)^c^	1.88 (0.61)	1.89 (0.61)	0.30	1.86 (0.59)	0.01
*Missing, % (n)*	*0.2 (17)*	*0.3 (24)*		*0.4 (214)*	

SHOT2022: Students’ Health and Wellbeing Study 2022; CIDI: Composite International Diagnostic Interview; HSCL-25: Hopkins Symptoms Checklist – 25 items version.

a Grand mean for the SHOT2022 sample aged 18–35.

b Compared with the CIDI responders group (p-values based on Chi-squared test (categorical variables) or t-test (continuous variables).

c Adjusted for sex.

The level of mental health problems, as assessed with the HSCL-25 in the SHOT2022 study, was slightly lower among students who completed the CIDI follow-up study (M = 1.88, SD = 0.61) compared to students who were invited, but did but did not respond (M = 1.89, SD = 0.61, Cohen’s *d* = 0.03). However, CIDI responders had slightly, but significantly, higher HSCL-25 score compared to the grand mean in the SHOT2022 sample (M = 1.86, SD = 0.59, Cohen’s *d* = 0.03). Also, the HSCL-25 score, assessed as part of the CIDI follow-up study, did not significantly differ between students who completed all CIDI modules (M = 1.88, SD = 0.57), and students who had missing data on one or more of the diagnostic modules (M = 1.88, SD = 0.74).

As detailed in Supplementary Table S1, participants who were invited to the CIDI study (n = 16,418) (irrespective of their response) were more likely to be female, had more financial difficulties, were more likely to be living alone, and had slightly higher HSCL-25 score (Cohen’s *d* = 0.03), compared with students who were not invited to the CIDI (n = 36,944).

### Prevalence of current disorder (30 days)

Overall, 39.7% (95% CI 38.6–40.9) of the female students and 25.7% (95% CI 24.2–27.4) of the male students had a current mental disorder (see Table 2, Table 3). The most common disorder was major depressive episode (females 17.1%, and males 10.8%), followed by GAD (females 16.0%, and males 8.2%). Less common anxiety disorders were social anxiety disorder (females 10.0%, and males 4.9%), specific phobias (females 9.6%, and males 3.0%), panic disorder (females 7.4%, and males 2.4%), and agoraphobia (females 2.0%, and males 0.7%). The opposite sex pattern was observed for substance-use disorders, including alcohol use disorder (females 5.6%, and males 7.7%) and drug use disorder (females 0.5%, and males 1.3%).

**Table 2:** Unweighted 30-days, 12-month, and lifetime prevalence^a^ of mental disorders among female students.

	30 days prevalence		12-month prevalence		Lifetime prevalence	
	% (95% CI)	Frequency (valid observations)	% (95% CI)	Frequency (valid observations)	% (95% CI)	Frequency (valid observations)
**Mood disorders**						
Major depressive episode	17.1% (16.2%, 17.9%)	1250 (7329)	35.3% (34.2%, 36.4%)	2572 (7289)	46.0% (44.9%, 47.2%)	3359 (7297)
**Anxiety disorders**						
Any anxiety disorder	29.9% (28.8%, 31.0%)	2092 (6999)	40.7% (39.5%, 41.9%)	2790 (6859)	48.4% (47.2%, 49.6%)	3333 (6886)
Generalized anxiety disorder	16.0% (15.2%, 16.9%)	1157 (7221)	23.8% (22.8%, 24.8%)	1625 (6837)	29.0% (27.9%, 30.1%)	1982 (6837)
Agoraphobia	2.0% (1.65%, 2.32%)	138 (7046)	2.9% (2.56%, 3.37%)	207 (7046)	3.7% (3.32%, 4.22%)	264 (7046)
Panic disorder	7.4% (6.76%, 7.98%)	529 (7197)	14.4% (13.6%, 15.2%)	1034 (7197)	19.4% (18.5%, 20.3%)	1396 (7197)
Social anxiety disorder	10.0% (9.27%, 10.7%)	697 (6998)	13.6% (12.8%, 14.4%)	949 (6998)	16.2% (15.4%, 17.1%)	1136 (6998)
Specific phobia	9.6% (8.96%, 10.4%)	679 (7047)	11.9% (11.1%, 12.7%)	838 (7047)	14.1% (13.3%, 14.9%)	994 (7047)
**Substance-use disorder**						
Any substance-use disorder	6.0% (5.41%, 6.55%)	406 (6815)	10.6% (9.90%, 11.4%)	724 (6819)	17.7% (16.8%, 18.6%)	1208 (6830)
Alcohol use disorder	5.6% (5.05%, 6.14%)	387 (6948)	7.7% (7.09%, 8.36%)	535 (6948)	16.1% (15.3%, 17.0%)	1119 (6948)
Drug use disorder	0.5% (0.39%, 0.76%)	37 (6817)	3.7% (3.27%, 4.18%)	252 (6817)	3.7% (3.27%, 4.18%)	252 (6817)
**Any disorder**	39.7% (38.6%, 40.9%)	2737 (6886)	57.3% (56.1%, 58.4%)	3977 (6945)	67.3% (66.1%, 68.3%)	4730 (7033)

a Prevalences are based on the number of female students with valid responses on the diagnostic section of interest.

**Table 3:** Unweighted 30-days, 12-month, and lifetime prevalence^a^ of mental disorders among male students.

	30 days prevalence		12-month prevalence		Lifetime prevalence	
	% (95% CI)	Frequency (valid observations)	% (95% CI)	Frequency (valid observations)	% (95% CI)	Frequency (valid observations)
**Mood disorders**						
Major depressive episode	10.8% (9.75%, 12.0%)	331 (3059)	24.7% (23.2%, 26.3%)	754 (3049)	34.0% (32.3%, 35.7%)	1037 (3050)
**Anxiety disorders**						
Any anxiety disorder	14.3% (13.0%, 15.6%)	423 (2967)	21.6% (20.1%, 23.1%)	630 (2922)	28.0% (26.3%, 29.6%)	819 (2930)
Generalized anxiety disorder	8.2% (7.30%, 9.30%)	250 (3032)	13.0% (11.8%, 14.3%)	384 (2949)	16.9% (15.6%, 18.3%)	498 (2949)
Agoraphobia	0.7% (0.47%, 1.13%)	22 (2983)	1.0% (0.69%, 1.45%)	30 (2983)	1.3% (0.94%, 1.80%)	39 (2983)
Panic disorder	2.4% (1.86%, 2.98%)	71 (3011)	6.0% (5.17%, 6.90%)	180 (3011)	8.7% (7.76%, 9.81%)	263 (3011)
Social anxiety disorder	4.9% (4.21%, 5.80%)	147 (2972)	7.1% (6.25%, 8.13%)	212 (2972)	8.8% (7.86%, 9.94%)	263 (2972)
Specific phobia	3.0% (2.45%, 3.71%)	90 (2982)	4.2% (3.48%, 4.95%)	124 (2982)	5.9% (5.13%, 6.86%)	177 (2982)
**Substance-use disorder**						
Any substance-use disorder	8.6% (7.62%, 9.69%)	251 (2918)	15.1% (13.8%, 16.5%)	441 (2918)	22.1% (20.6%, 23.6%)	645 (2920)
Alcohol use disorder	7.7% (6.77%, 8.73%)	228 (2963)	10.4% (9.33%, 11.6%)	308 (2963)	18.6% (17.3%, 20.1%)	552 (2963)
Drug use disorder	1.3% (0.91%, 1.76%)	37 (2916)	6.2% (5.37%, 7.16%)	181 (2916)	6.2% (5.37%, 7.16%)	181 (2916)
**Any disorder**	25.7% (24.2%, 27.4%)	751 (2918)	42.5% (40.7%, 44.3%)	1240 (2916)	53.6% (51.7%, 55.4%)	1573 (2937)

a Prevalences are based on the number of female students with valid responses on the diagnostic section of interest.

### 12-Months prevalence

In all, 57.3% (56.1%–58.4%) of the female students and 42.5% (40.7%–44.3%) of the male students fulfilled the criteria for a 12-month mental disorder. For females, the most common disorder was major depressive episode (35.3%), followed by GAD (23.8%), panic disorder (14.4%) and social anxiety disorder (13.6%). A similar pattern was observed for males, but with lower prevalence (major depressive episode: 24.7%, GAD: 13.0%, social anxiety disorder (7.1%), and panic disorder (6%). The 12-month prevalence of alcohol use disorder and drug use disorder was 7.7% and 3.7%, respectively, among females, and 10.4% and 6.2%, respectively, among males.

### Lifetime prevalence

Sixty-seven percent of the female students fulfilled the diagnostic criteria for a lifetime disorder, while the corresponding prevalence among male students was 53.6%. Major depressive episode was also the most common disorder when examining the lifetime prevalence of mental disorders, with 46% of the females and 34.0% of the males fulfilling the diagnostic criteria. The lifetime prevalence of GAD was relatively similar for females and males (29% and 28%, respectively). The lifetime prevalence of the less common anxiety disorder is detailed in Table 2, Table 3. In terms of alcohol use disorder, the lifetime prevalence for females and males were 16.1% and 18.6%, respectively.

### Age differences

For most disorders, there was a U-shaped pattern regarding age differences in the 12-month prevalence of mental disorders. For example, the prevalence of major depressive episode among female students aged 18–22 years was 35.6%, compared to 33.5% and 37.6% among female students aged 23–25 years and 26–35 years, respectively. The similar pattern was observed for male students. However, for GAD and drug use disorder, the prevalence increased with advancing age (see Table 4, Table 5 for details). Similar age patterns were also present for 30-days and lifetime disorders (see Supplementary Tables S2–S5).

**Table 4:** Unweighted 12-month prevalence^a^ of mental disorders among females by age group.

	Age 18–22 years		Age 23–25 years		Age 26–35 years	
	% (95% CI)	Frequency (valid observations)	% (95% CI)	Frequency (valid observations)	% (95% CI)	Frequency (valid observations)
**Mood disorders**						
Major depressive episode	35.6% (33.7%, 37.5%)	883 (2481)	33.5% (31.8%, 35.2%)	967 (2888)	37.6% (35.4%, 39.8%)	722 (1920)
**Anxiety disorders**						
Any anxiety disorder	40.9% (38.9%, 42.9%)	951 (2327)	38.5% (36.7%, 40.4%)	1047 (2718)	43.7% (41.4%, 46.0%)	792 (1814)
Generalized anxiety disorder	21.7% (20.1%, 23.5%)	503 (2313)	22.7% (21.2%, 24.4%)	616 (2711)	27.9% (25.9%, 30.0%)	506 (1813)
Agoraphobia	3.2% (2.52%, 3.97%)	76 (2399)	2.4% (1.85%, 3.01%)	66 (2794)	3.5% (2.74%, 4.48%)	65 (1853)
Panic disorder	15.3% (13.9%, 16.8%)	374 (2445)	13.7% (12.4%, 15.0%)	390 (2852)	14.2% (12.7%, 15.9%)	270 (1900)
Social anxiety disorder	13.9% (12.6%, 15.4%)	331 (2376)	12.4% (11.2%, 13.7%)	345 (2783)	14.8% (13.3%, 16.6%)	273 (1839)
Specific phobia	12.0% (10.7%, 13.3%)	287 (2400)	11.2% (10.0%, 12.4%)	312 (2796)	12.9% (11.4%, 14.5%)	239 (1851)
**Substance-use disorder**						
Any substance-use disorder	10.9% (9.65%, 12.2%)	251 (2308)	9.6% (8.56%, 10.8%)	261 (2710)	11.8% (10.3%, 13.4%)	212 (1801)
Alcohol use disorder	8.7% (7.61%, 9.93%)	205 (2356)	7.5% (6.56%, 8.56%)	207 (2760)	6.7% (5.63%, 7.98%)	123 (1832)
Drug use disorder	2.8% (2.20%, 3.60%)	65 (2307)	2.8% (2.23%, 3.52%)	76 (2710)	6.2% (5.12%, 7.40%)	111 (1800)
**Any disorder**	58.0% (56.0%, 60.0%)	1363 (2350)	55.0% (53.1%, 56.8%)	1512 (2751)	59.8% (57.5%, 62.0%)	1102 (1844)

a Prevalences are based on the number of female students with valid responses on the diagnostic section of interest.

**Table 5:** Unweighted 12-month prevalence^a^ of mental disorders among males by age group.

	Age 18–22 years		Age 23–25 years		Age 26–35 years	
	% (95% CI)	Frequency (valid observations)	% (95% CI)	Frequency (valid observations)	% (95% CI)	Frequency (valid observations)
**Mood disorders**						
Major depressive episode	23.2% (20.5%, 26.2%)	201 (865)	21.9% (19.6%, 24.4%)	261 (1191)	29.4% (26.6%, 32.4%)	292 (993)
**Anxiety disorders**						
Any anxiety disorder	18.1% (15.6%, 20.9%)	150 (829)	19.2% (17.0%, 21.7%)	221 (1149)	27.4% (24.6%, 30.4%)	259 (944)
Generalized anxiety disorder	8.9% (7.15%, 11.1%)	75 (838)	11.3% (9.60%, 13.3%)	131 (1155)	18.6% (16.2%, 21.3%)	178 (956)
Agoraphobia	0.8% (0.36%, 1.77%)	7 (850)	0.7% (0.32%, 1.40%)	8 (1171)	1.6% (0.91%, 2.62%)	15 (962)
Panic disorder	5.7% (4.31%, 7.56%)	49 (855)	5.5% (4.30%, 6.99%)	65 (1183)	6.8% (5.32%, 8.60%)	66 (973)
Social anxiety disorder	7.1% (5.49%, 9.08%)	60 (847)	5.4% (4.20%, 6.89%)	63 (1167)	9.3% (7.56%, 11.4%)	89 (958)
Specific phobia	3.9% (2.73%, 5.48%)	33 (849)	4.4% (3.37%, 5.82%)	52 (1171)	4.1% (2.94%, 5.55%)	39 (962)
**Substance-use disorder**						
Any substance-use disorder	14.6% (12.3%, 17.3%)	122 (834)	14.0% (12.1%, 16.2%)	160 (1139)	16.8% (14.5%, 19.4%)	159 (945)
Alcohol use disorder	11.4% (9.35%, 13.8%)	96 (844)	10.5% (8.83%, 12.4%)	122 (1162)	9.4% (7.67%, 11.5%)	90 (957)
Drug use disorder	4.4% (3.19%, 6.14%)	37 (832)	5.1% (3.92%, 6.58%)	58 (1139)	9.1% (7.38%, 11.2%)	86 (945)
**Any disorder**	41.8% (38.4%, 45.2%)	343 (821)	39.1% (36.2%, 42.0%)	448 (1147)	47.4% (44.1%, 50.6%)	449 (948)

a Prevalences are based on the number of male students with valid responses on the diagnostic section of interest.

## Discussion

This is the first study to use the newly developed self-administered electronic version of the CIDI 5.0, enabling the recruitment of a large national sample of college and university students at comparatively low cost. Based on a sample of around 10,000 students, the current study found a disturbingly high prevalence of several mental disorders. In all, 40% of the female students and 26% of the male students were identified with a current mental disorder, and the 12-month and lifetime prevalence was, as expected, even higher.

The main distinction between the current study and previous research lies in the mental health measures employed. While scoring tools and questionnaires mainly evaluate short-term periods of mental health problems, diagnostic tools that evaluate mental disorders, such as CIDI, also include criteria assessing functional impairment, disability, and symptom duration and intensity, which raises the threshold for identifying cases. Although the former may be effective in detecting fluctuations in mental distress, these fluctuations do not necessarily equate to an escalation in clinically significant mental disorders. Surprisingly few studies have used structured diagnostic interviews, with the latest published studies now being more than a decade old.^2^^,^^3^ However, the Dutch NEMESIS-3 study,^11^^,^^12^ which monitors the prevalence of mental disorders in the Dutch general population, provides some important insights to the field. Using CIDI 3.0 (face-to-face interview), the 12-month prevalence of any mental disorder was found to be 40% and 35% among young adults aged 18–24 years, and 25–35 years, respectively. The corresponding lifetime estimates were 50% and 59%, respectively. The current prevalence estimates are also higher than what was reported in a follow-up study of mental disorders in the HUNT study from 2020.^32^ Interviewing a total of 2154 participants from the general population using the CIDI 5.0, the 30-day prevalence of a mental disorder among participants aged 20–29 years was estimated to be 25.5% just before the outbreak of the COVID-19 pandemic. While the prevalence estimates from NEMESIS-3 and HUNT are still lower than those observed in the current study of college and university students, all three studies clearly demonstrate that mental disorders have become highly prevalent among young adults. And while brief survey questionnaires assessing symptoms of anxiety and/or depression may not be sufficient to identify mental disorders per se, some self-report instruments, such as the GAD-7^33^ and PHQ-9,^34^ also include a functional rating scale to gauge the impact of the reported symptoms, which make them relevant in this context. For example, in a Canadian study^16^ of 1530 first-year university students, 36% and 39%, respectively, displayed clinically significant symptoms of depression and anxiety. As such, the current evidence base is highly concerning, demonstrating that a substantial minority of college and university students are suffering from a mental disorder severe enough to require intervention. We also know that approximately half of the people who suffer from a major depressive episode for the first time, experience recurrences, and new episodes.^35^ While some studies^36^ do show an increase in help-seeking behaviour, there are multiple indications that only a minority of young people seek and receive sufficient help for their mental health problems.^37^^,^^38^ Given the negative impact of mental disorders on e.g., academic performance,^16^ which in turn can affect future employment opportunities, it is imperative to investigate this issue further. Both policy makers, educational institutions, and student welfare organizations need to ensure that the necessary support mechanisms are sufficiently scaled.

The observed high prevalence can potentially stem from multiple causes. From a methodological standpoint, it has been suggested that recent changes in self-reporting caused by decreasing stigma of mental health, may in turn boost the reporting of mental health problems per se.^39^ However, recent findings from the UK using time trend data from 2009 to 2019 found no support for the argument that changes in public stigma could explain the sharp increase in self-reported mental health problems over the last decade.^40^ Another explanation may be related to the growing proportion of young adults who are pursuing higher education. Consequently, the student population is becoming more diverse and may include a larger proportion of individuals who lack the necessary resources to handle the demands of student life or who experience mental health problems more frequently. Lastly, there are several risk factors that possibly may have contributed to the decline in the mental health of young adults and students. These include an increase in loneliness,^41^ perfectionism.^42^ individualism and focus on appearance,^43^ as well as educational expectations, the potential negative impact of social media, and changes in drug use, particularly cannabis use.^44^ Additionally, one may speculate if there have been any changes in the susceptibility to risk factors and whether the younger generation possesses the necessary coping skills to handle the normal stress and pressures of life. However, there is limited existing evidence to support an increased vulnerability among adolescents,^45^ and none of these factors have been thoroughly investigated within the context of higher education. It should also be noted that the current study was conducted more than a year after the removal of the many restrictions caused by the COVID-19 pandemic in Norway. While especially students showed a sharp deterioration in mental health in the initial phase of the pandemic,^19^^,^^20^ the current study demonstrates that the mental health of college and university students remains an issue of concern.

The strengths of the study include the use of data assessed through a standardized and validated instrument (the CIDI). However, the fact that the current study used a newly developed self-administered electronic questionnaire version of CIDI 5.0, rather than the traditional face-to-face or telephone versions, poses some new challenges. Although previous studies using CIDI 5.0 have found no differences in prevalence estimates comparing face-to-face to telephone interviews,^32^ no studies have so far validated this self-administered version of CIDI against the face-to-face CIDI or a semi-structured clinical diagnostic assessment by a mental health professional. A recent study comparing face-to-face and web modes on self-reported psychological functioning, found that individuals who responded face-to-face reported slightly fewer depressive symptoms compared to those who responded over the web (Cohen’s *d* = 0.25),^46^ suggesting that social desirability bias may influence responses achieved in the presence of an interviewer. However, which mode yields the most valid estimate may be debatable, as previous studies have also found that young and well-educated respondents are particularly prone to under-report symptoms of anxiety and depression when being interviewed.^47^ Thus, the potential administration mode effects should be considered when interpreting results obtained through different survey modes. Another limitation related to the SHOT2022 study, is that we do not know whether non-respondents of the SHOT2022 are more or less likely to have mental disorders than respondents. While it has been shown that non-participants of health surveys generally have worse health than participants,^48^ people may also be more prone to participate in a survey if the topic seems relevant to them personally.^49^ However, we found only marginal differences in the level of mental health problems (assessed with the HSCL-25 in the SHOT2022) between responders and non-responders of the CIDI study, and the extent of missing data within the CIDI survey was not significantly associated with the HSCL-25 score. Moreover, the only information we had on non-responders was the age- and sex distribution, which did not differ from SHOT2022 responders. We were also unable to assess how representative the current study sample was with respect to the reference population (that is, Norwegian higher education students) in terms of demographical and educational aspects. However, both the response rates and the HSCL-25 scores were relatively similar across the health regions in Norway, suggesting that the representativeness in terms of geography was reasonable. Finally, we did not assess all diagnostic categories included in the CIDI in the current study. The following disorders/diagnostic categories were omitted to minimize the burden on participating students, and thus increase the response rate: bipolar disorders, obsessive-compulsive disorders, post-traumatic stress disorder (PTSD), attention-deficit/hyperactivity disorder (ADHD), and personality disorders. Also, eating disorders are not included in the CIDI, but would be interesting to examine, as recent studies have indicated a sharp increase in eating disorders in recent years.^50^ As such, future studies should also examine to the prevalence of these disorders among college and university students.

## Contributors

Børge Sivertsen (BS) and Kari-Jussie Lønning (KJL) contributed with initiation, planning and design of the SHOT2022 data-collection. Ronald C. Kessler (RCK) led the development of the CIDI. BS, Ann Kristin Skrindo Knudsen (AKSK), Anne Reneflot (AR), Benedicte Kirkøen (BK) and Bengt Oscar Lagerstrøm (BOL) contributed with initiation, planning and design of the CIDI data-collection. BOL led the data-collection, while BS coordinated the study and led the writing of the manuscript. AKSK and Jens Christoffer Skogen (JCS) conducted the statistical analyses. AR conducted the literature review. All authors contributed with input on design and analytical plan, interpretation of results, writing of the first draft, and critical revision of the manuscript and analyses. All authors approved the submission.

## Data sharing statement

Norwegian data protection regulations and GDPR impose restrictions on sharing of individual participant data. However, researchers may gain access to survey participant data by contacting the publication committee (borge.sivertsen@fhi.no). Approval from the Norwegian Regional Committee for Medical and Health Research Ethics (https://helseforskning.etikkom.no) is a pre-requirement for access to the data. The dataset is administrated by the NIPH, and guidelines for access to data are found at https://www.fhi.no/en/more/access-to-data. Analytic codes for the analyses are available upon request to the corresponding author.

## Declaration of interests

In the past 3 years, RCK was a consultant for Cambridge Health Alliance, Canandaigua VA Medical Center, Holmusk, Partners Healthcare, Inc., RallyPoint Networks, Inc., and Sage Therapeutics. He has stock options in Cerebral Inc., Mirah, PYM, Roga Sciences and Verisense Health. All other authors declare no competing interests.

## Appendix A Supplementary data

Supplemenatry Tables S1–S5

Norwegian abstract
