# Supplementary material for: Prevalence of mental disorders among Norwegian college and university students: a population-based cross-sectional analysis
Source: Lancet Reg Health Eur. 2023 Sep 19;34:100732. doi: 10.1016/j.lanepe.2023.100732 (PMC10624983; doi:10.1016/j.lanepe.2023.100732)
Supplement: Supplemenatry Tables S1–S5 [file mmc1.docx]

| **Supplementary Table 1.** Sociodemographic and clinical characteristics of participants in SHOT2022 who were invited (n=16,418) versus those who were not invited (n=36,944) to the CIDI follow-up study | | | |
| --- | --- | --- | --- |
| **Characteristic** | **Invited to CIDI** (n=16,418) | **Not invited to CIDI** (n=36,944) | **p-value^#^** |
| Age, mean (SD) | 24.03 (3.26) | 23.97 (3,25) | .05 |
| Age group, % (n) |  |  | .57 |
| 18-22 years | 46.0 (7,528) | 46.5 (17,179) |  |
| 23-25 years | 32.9 (5,399) | 32.7 (12,083) |  |
| 26-35 years | 21.2 (3,491) | 20.8 (7,682) |  |
| Sex, % (n) |  |  | <.001 |
| Women | 70.4 (11,564) | 64.4 (23,802) |  |
| Men | 29.6 (4,854) | 35.6 (13,142) |  |
| Marital status, % (n) |  |  | .86 |
| Single | 50.9 (8,361) | 51.0 (18,844) |  |
| Boy-/girlfriend | 23.0 (3,769) | 22.7 (8,384) |  |
| Cohabitant | 22.5 (3,701) | 22.6 (8,345) |  |
| Married/registered partner | 3.2 (518) | 3.1 (1,152) |  |
| *Missing* | *0.4 % (69)* | *0.6 (219)* |  |
| Financial difficulties, % (n) |  |  | .04 |
| Never | 53.7 (8,809) | 54.2 (20,008) |  |
| Rarely | 20.2 (3,309) | 21.1 (7,803) |  |
| Sometimes | 19.1 (3,142) | 18.2 (6,739 |  |
| Often | 6.7 (1,095) | 5.8 (2,146) |  |
| *Missing* | *0.4 (63)* | *0.7 (248)* |  |
| Self and/or parent(s) born abroad, % (n) |  |  | .94 |
| Born in Norway | 80.9 (13,282) | 80.6 (29,767) |  |
| Born outside Norway | 10.3 (1,699) | 10.4 (3,848) |  |
| *Missing* | *8.8 (1437)* | *9.0 (3,329)* |  |
| Accommodation status, % (n) |  |  | .09 |
| Living alone | 20.1 (3,311) | 19.2 (7,110) |  |
| Living with partner | 26.6 (4,370) | 26.7 (9,846) |  |
| Living with friends | 45.0 (7,357) | 44.7 (16,499) |  |
| Living with parents | 7.9 (1,328) | 8.9 (3,370) |  |
| *Missing* | *0.3 (52)* | *0.6 (219)* |  |
| Maternal education, % (n) |  |  | .30 |
| Primary | 4.7 (770) | 4.4 (1,634) |  |
| Secondary | 27.4 (4,499) | 27.6 (10,206) |  |
| College/university | 65.1 (10,690) | 64.0 (23,634) |  |
| *Missing* | *2.8 (459)* | *4.0 (1,470)* |  |
| Paternal education, % (n) |  |  | .51 |
| Primary | 6.1 (1,006) | 5.9 (2,172) |  |
| Secondary | 35.0 (5,745) | 35.1 (12,983) |  |
| College/university | 53.8 (8,830) | 53.1 (19,626) |  |
| Missing | 5.1 (837) | 5.9 (2,163) |  |
| HSCL-25, Mean (SD) ^$^ | 1.88 (0.61) | 1.86 (0.59) | <.001 |
| *Missing, % (n)* | *0.3 (41)* | *0.5 (173)* |  |
| SHOT2022: Students’ Health and Wellbeing Study 2022; CIDI: Composite International Diagnostic Interview; HSCL-25 Hopkins Symptoms Checklist – 25 items version **^#^** p-values based on Chi-squared test (categorical variables) or t-test (continuous variables) | | | |

| **Supplementary Table 2:** Unweighted 30-days prevalence^1^ of mental disorders among females by age group. | | | | | | |
| --- | --- | --- | --- | --- | --- | --- |
|  | **Age 18-22 years** | | **Age 23-25 years** | | **Age 26-35 years** | |
|  | % (95% CI) | Frequency  (valid observations) | % (95% CI) | Frequency  (valid observations) | % (95% CI) | Frequency  (valid observations) |
| **Mood disorders** |  |  |  |  |  |  |
| Major depressive episode | 17.0% (15.5%, 18.5%) | 422 (2,488) | 16.1% (14.8%, 17.5%) | 467 (2,908) | 18.7% (17.0%, 20.5%) | 361 (1,933) |
| **Anxiety disorders** |  |  |  |  |  |  |
| Any anxiety disorder | 31.1% (29.3%, 33.0%) | 739 (2,376) | 27.4% (25.8%, 29.1%) | 764 (2,784) | 32.0% (29.9%, 34.2%) | 589 (1,839) |
| Generalized anxiety disorder | 15.4% (14.0%, 16.9%) | 380 (2,461) | 15.0% (13.7%, 16.4%) | 430 (2,862) | 18.3% (16.6%, 20.1%) | 347 (1,898) |
| Agoraphobia | 2.2% (1.67%, 2.90%) | 53 (2,399) | 1.6% (1.19%, 2.17%) | 45 (2,794) | 2.2% (1.57%, 2.96%) | 40 (1,853) |
| Panic disorder | 8.1% (7.02%, 9.22%) | 197 (2,445) | 6.8% (5.95%, 7.84%) | 195 (2,852) | 7.2% (6.11%, 8.49%) | 137 (1,900) |
| Social anxiety disorder | 10.2% (9.01%, 11.5%) | 242 (2,376) | 9.3% (8.27%, 10.5%) | 259 (2,783) | 10.7% (9.30%, 12.2%) | 196 (1,839) |
| Specific Phobia | 9.8% (8.61%, 11.0%) | 234 (2,400) | 8.6% (7.62%, 9.74%) | 241 (2,796) | 11.0% (9.65%, 12.6%) | 204 (1,851) |
| **Substance-use disorder** |  |  |  |  |  |  |
| Any substance-use disorder | 6.9% (5.87%, 7.98%) | 158 (2,306) | 6.1% (5.20%, 7.03%) | 164 (2,710) | 4.7% (3.76%, 5.77%) | 84 (1,799) |
| Alcohol use disorder | 6.5% (5.51%, 7.54%) | 152 (2,356) | 5.7% (4.87%, 6.64%) | 157 (2,760) | 4.3% (3.40%, 5.31%) | 78 (1,832) |
| Drug use disorder | 0.6% (0.35%, 1.04%) | 14 (2,307) | 0.5% (0.29%, 0.89%) | 14 (2,710) | 0.5% (0.24%, 0.98%) | 9 (1,800) |
| **Any disorder** | 41.6% (39.6%, 43.6%) | 971 (2,334) | 37.8% (36.0%, 39.7%) | 1,036 (2,740) | 40.3% (38.0%, 42.6%) | 730 (1,812) |
| ^1^ Prevalences are based on the number of female students with valid responses on the diagnostic section of interest | | | | | | |

| **Supplementary Table 3:** Unweighted 30-days prevalence^1^ of mental disorders among males by age group. | | | | | | |
| --- | --- | --- | --- | --- | --- | --- |
|  | **Age 18-22 years** | | **Age 23-25 years** | | **Age 26-35 years** | |
|  | % (95% CI) | Frequency  (valid observations) | % (95% CI) | Frequency  (valid observations) | % (95% CI) | Frequency  (valid observations) |
| **Mood disorders** |  |  |  |  |  |  |
| Major depressive episode | 8.3% (6.60%, 10.4%) | 72 (866) | 9.2% (7.64%, 11.0%) | 110 (1,197) | 15.0% (12.8%, 17.4%) | 149 (996) |
| **Anxiety disorders** |  |  |  |  |  |  |
| Any anxiety disorder | 11.6% (9.57%, 14.0%) | 98 (844) | 12.2% (10.4%, 14.3%) | 142 (1,163) | 19.1% (16.7%, 21.7%) | 183 (960) |
| Generalized anxiety disorder | 5.9% (4.48%, 7.76%) | 51 (862) | 6.6% (5.27%, 8.19%) | 78 (1,185) | 12.3% (10.3%, 14.5%) | 121 (985) |
| Agoraphobia | 0.7% (0.29%, 1.61%) | 6 (850) | 0.4% (0.16%, 1.05%) | 5 (1,171) | 1.1% (0.60%, 2.10%) | 11 (962) |
| Panic disorder | 2.1% (1.29%, 3.37%) | 18 (855) | 2.4% (1.61%, 3.45%) | 28 (1,183) | 2.6% (1.70%, 3.83%) | 25 (973) |
| Social anxiety disorder | 4.7% (3.44%, 6.43%) | 40 (847) | 3.7% (2.71%, 4.97%) | 43 (1,167) | 6.7% (5.22%, 8.50%) | 64 (958) |
| Specific Phobia | 2.6% (1.67%, 3.96%) | 22 (849) | 3.2% (2.34%, 4.47%) | 38 (1,171) | 3.1% (2.15%, 4.48%) | 30 (962) |
| **Substance-use disorder** |  |  |  |  |  |  |
| Any substance-use disorder | 10.1% (8.15%, 12.4%) | 84 (834) | 8.3% (6.83%, 10.1%) | 95 (1,139) | 7.6% (6.05%, 9.55%) | 72 (945) |
| Alcohol use disorder | 9.0% (7.20%, 11.2%) | 76 (844) | 7.7% (6.23%, 9.38%) | 89 (1,162) | 6.6% (5.13%, 8.39%) | 63 (957) |
| Drug use disorder | 1.2% (0.61%, 2.28%) | 10 (832) | 1.2% (0.70%, 2.11%) | 14 (1,139) | 1.4% (0.77%, 2.41%) | 13 (945) |
| **Any disorder** | 24.1% (21.3%, 27.2%) | 199 (825) | 23.2% (20.8%, 25.8%) | 265 (1,143) | 30.2% (27.3%, 33.3%) | 287 (950) |
| ^1^ Prevalences are based on the number of male students with valid responses on the diagnostic section of interest | | | | | | |

| **Supplementary Table 4:** Unweighted lifetime prevalence^1^ of mental disorders among females by age group. | | | | | | |
| --- | --- | --- | --- | --- | --- | --- |
|  | **Age 18-22 years** | | **Age 23-25 years** | | **Age 26-35 years** | |
|  | % (95% CI) | Frequency  (valid observations) | % (95% CI) | Frequency  (valid observations) | % (95% CI) | Frequency  (valid observations) |
| **Mood disorders** |  |  |  |  |  |  |
| Major depressive episode | 43.4% (41.4%, 45.4%) | 1,077 (2,482) | 44.2% (42.4%, 46.1%) | 1,279 (2,892) | 52.2% (49.9%, 54.4%) | 1,003 (1,923) |
| **Anxiety disorders** |  |  |  |  |  |  |
| Any anxiety disorder | 46.7% (44.7%, 48.8%) | 1,089 (2,331) | 46.4% (44.5%, 48.2%) | 1,265 (2,729) | 53.6% (51.3%, 55.9%) | 979 (1,826) |
| Generalized anxiety disorder | 25.2% (23.5%, 27.0%) | 583 (2,313) | 27.8% (26.2%, 29.6%) | 755 (2,711) | 35.5% (33.3%, 37.8%) | 644 (1,813) |
| Agoraphobia | 3.5% (2.78%, 4.29%) | 83 (2,399) | 3.2% (2.61%, 3.96%) | 90 (2,794) | 4.9% (3.99%, 6.02%) | 91 (1,853) |
| Panic disorder | 19.5% (18.0%, 21.1%) | 477 (2,445) | 18.2% (16.8%, 19.7%) | 520 (2,852) | 21.0% (19.2%, 22.9%) | 399 (1,900) |
| Social anxiety disorder | 16.3% (14.9%, 17.9%) | 388 (2,376) | 14.8% (13.5%, 16.2%) | 412 (2,783) | 18.3% (16.5%, 20.1%) | 336 (1,839) |
| Specific Phobia | 13.7% (12.4%, 15.2%) | 329 (2,400) | 13.1% (11.8%, 14.4%) | 365 (2,796) | 16.2% (14.6%, 18.0%) | 300 (1,851) |
| **Substance-use disorder** |  |  |  |  |  |  |
| Any substance-use disorder | 15.5% (14.1%, 17.1%) | 359 (2,311) | 16.1% (14.8%, 17.6%) | 438 (2,713) | 22.8% (20.9%, 24.8%) | 411 (1,806) |
| Alcohol use disorder | 14.2% (12.8%, 15.7%) | 335 (2,356) | 14.8% (13.5%, 16.2%) | 408 (2,760) | 20.5% (18.7%, 22.5%) | 376 (1,832) |
| Drug use disorder | 2.8% (2.20%, 3.60%) | 65 (2,307) | 2.8% (2.23%, 3.52%) | 76 (2,710) | 6.2% (5.12%, 7.40%) | 111 (1,800) |
| **Any disorder** | 65.4% (63.4%, 67.3%) | 1,548 (2,368) | 65.6% (63.8%, 67.3%) | 1,830 (2,790) | 72.1% (70.0%, 74.1%) | 1,352 (1,875) |
| ^1^ Prevalences are based on the number of female students with valid responses on the diagnostic section of interest | | | | | | |

| **Supplementary Table 5:** Unweighted lifetime prevalence^1^ of mental disorders among males by age group. | | | | | | |
| --- | --- | --- | --- | --- | --- | --- |
|  | **Age 18-22 years** | | **Age 23-25 years** | | **Age 26-35 years** | |
|  | % (95% CI) | Frequency  (valid observations) | % (95% CI) | Frequency  (valid observations) | % (95% CI) | Frequency  (valid observations) |
| **Mood disorders** |  |  |  |  |  |  |
| Major depressive episode | 30.6% (27.6%, 33.8%) | 265 (865) | 30.1% (27.5%, 32.8%) | 358 (1,191) | 41.6% (38.6%, 44.8%) | 414 (994) |
| **Anxiety disorders** |  |  |  |  |  |  |
| Any anxiety disorder | 24.5% (21.7%, 27.6%) | 204 (832) | 24.9% (22.4%, 27.5%) | 286 (1,150) | 34.7% (31.7%, 37.8%) | 329 (948) |
| Generalized anxiety disorder | 12.5% (10.4%, 15.0%) | 105 (838) | 14.7% (12.8%, 16.9%) | 170 (1,155) | 23.3% (20.7%, 26.2%) | 223 (956) |
| Agoraphobia | 1.1% (0.52%, 2.08%) | 9 (850) | 1.1% (0.62%, 1.94%) | 13 (1,171) | 1.8% (1.07%, 2.88%) | 17 (962) |
| Panic disorder | 7.5% (5.85%, 9.51%) | 64 (855) | 7.9% (6.42%, 9.58%) | 93 (1,183) | 10.9% (9.04%, 13.1%) | 106 (973) |
| Social anxiety disorder | 9.1% (7.28%, 11.3%) | 77 (847) | 6.4% (5.12%, 8.03%) | 75 (1,167) | 11.6% (9.66%, 13.8%) | 111 (958) |
| Specific Phobia | 5.5% (4.14%, 7.35%) | 47 (849) | 6.2% (4.95%, 7.81%) | 73 (1,171) | 5.9% (4.56%, 7.66%) | 57 (962) |
| **Substance-use disorder** |  |  |  |  |  |  |
| Any substance-use disorder | 17.9% (15.4%, 20.7%) | 149 (834) | 21.5% (19.2%, 24.0%) | 245 (1,139) | 26.5% (23.7%, 29.5%) | 251 (947) |
| Alcohol use disorder | 15.0% (12.7%, 17.7%) | 127 (844) | 18.8% (16.7%, 21.2%) | 219 (1,162) | 21.5% (19.0%, 24.3%) | 206 (957) |
| Drug use disorder | 4.4% (3.19%, 6.14%) | 37 (832) | 5.1% (3.92%, 6.58%) | 58 (1,139) | 9.1% (7.38%, 11.2%) | 86 (945) |
| **Any disorder** | 49.8% (46.4%, 53.3%) | 413 (829) | 50.3% (47.3%, 53.2%) | 579 (1,152) | 60.8% (57.6%, 63.9%) | 581 (956) |
| ^1^ Prevalences are based on the number of male students with valid responses on the diagnostic section of interest | | | | | | |
