# Supplementary material for: Prevalence of mental disorders among Norwegian college and university students: a population-based cross-sectional analysis
Source: Lancet Reg Health Eur. 2023 Sep 19;34:100732. doi: 10.1016/j.lanepe.2023.100732 (PMC10624983; doi:10.1016/j.lanepe.2023.100732)
Supplement: Norwegian abstract [file mmc2.docx]

*This translation in Norwegian was submitted by the authors and we reproduce it as supplied. It has not been peer reviewed. Our editorial processes have only been applied to the original abstract in English, which should serve as reference for this manuscript*

**SAMMENDRAG**

*Bakgrunn:* Selvrapporterte data har vist en skarp økning i psykiske helseproblemer blant høgskole- og universitetsstudenter de siste årene, men nøyaktige forekomsttall for psykiske lidelser mangler. Den nåværende studien brukte en validert psykiatrisk diagnostisk undersøkelse, utviklet til en elektronisk selvadministrert versjon, for å undersøke forekomsten av vanlige psykiske lidelser i et stort nasjonalt utvalg av høgskole- og universitetsstudenter i Norge.

*Metode:* Deltakere (i alderen 18-35 år) fra Studentenes Helse- og Trivselsundersøkelse (SHOT) i 2022 ble rekruttert til en oppfølgingsundersøkelse om psykiske lidelser fra januar til februar 2023 (n= 10,460). Nåværende (30 dages), 12-måneders og livstidsforekomst av vanlige psykiske lidelser ble undersøkt ved hjelp av en selvadministrert versjon av Composite International Diagnostic Interview (CIDI 5.0).

*Resultater:* Forekomsten av en nåværende psykisk lidelse var høy for både kvinner (39,7%, 95% CI 38,6-40,9) og menn (25,7%, 95% CI 24,2-27,4). De vanligste lidelsene var alvorlig depressiv episode (kvinner 17,1% og menn 10,8%) og generalisert angstlidelse (kvinner 16,0% og menn 8,2%), mens 5,6% og 7,7% av kvinnelige og mannlige studenter henholdsvis oppfylte kriteriene for en alkoholbrukslidelse. Forekomstestimatene for 12 måneder og livstid var, som forventet, enda høyere.

*Konklusjon:* Funnene viser en bekymringsfullt høy forekomst av flere psykiske lidelser blant norske høgskole- og universitetsstudenter. Implikasjoner og mulige metodiske og kontekstuelle forklaringer på disse funnene diskuteres.
